# Nipah Virus Detection at Bat Roosts after Spillover Events, Bangladesh, 2012–2019

**DOI:** 10.3201/eid2807.212614

**Published:** 2022-07

**Authors:** Clifton D. McKee, Ausraful Islam, Mohammed Ziaur Rahman, Salah Uddin Khan, Mahmudur Rahman, Syed M. Satter, Ariful Islam, Claude Kwe Yinda, Jonathan H. Epstein, Peter Daszak, Vincent J. Munster, Peter J. Hudson, Raina K. Plowright, Stephen P. Luby, Emily S. Gurley

**Affiliations:** Bloomberg School of Public Health, Johns Hopkins University, Baltimore, Maryland, USA (C.D. McKee, E.S. Gurley);; icddr,b, Dhaka, Bangladesh (Ausraful Islam, M.Z. Rahman, S.U. Khan, M. Rahman, S.M. Satter, E.S. Gurley);; Institute of Epidemiology, Disease Control and Research, Dhaka, Bangladesh (M.Z. Rahman);; Global Health Development/Eastern Mediterranean Public Health Network, Amman, Jordan (M.Z. Rahman);; Deakin University, Geelong, Victoria, Australia (Ariful Islam);; EcoHealth Alliance, New York, New York, USA (Ariful Islam, J.H. Epstein, P. Daszak);; National Institute of Allergy and Infectious Diseases, National Institutes of Health, Hamilton, Montana, USA (C.K. Yinda, V.J. Munster);; Pennsylvania State University, State College, Pennsylvania, USA (P.J. Hudson);; Montana State University, Bozeman, Montana, USA (R.K. Plowright);; Stanford University, Stanford, California, USA (S.P. Luby)

**Keywords:** Nipah virus, surveillance, zoonotic pathogens, Henipavirus, Chiroptera, Pteropodidae, spillover, cross-species transmission, zoonoses, viruses, Bangladesh

## Abstract

Knowledge of the dynamics and genetic diversity of Nipah virus circulating in bats and at the human-animal interface is limited by current sampling efforts, which produce few detections of viral RNA. We report a series of investigations at *Pteropus medius* bat roosts identified near the locations of human Nipah cases in Bangladesh during 2012–2019. Pooled bat urine was collected from 23 roosts; 7 roosts (30%) had >1 sample in which Nipah RNA was detected from the first visit. In subsequent visits to these 7 roosts, RNA was detected in bat urine up to 52 days after the presumed exposure of the human case-patient, although the probability of detection declined rapidly with time. These results suggest that rapidly deployed investigations of Nipah virus shedding from bat roosts near human cases could increase the success of viral sequencing compared with background surveillance and could enhance understanding of Nipah virus ecology and evolution.

Nipah virus is a paramyxovirus (genus *Henipavirus*) that has caused outbreaks of neurologic and respiratory disease in humans and livestock in Bangladesh, India, Malaysia, Singapore, and the Philippines (*1*–*4*). The primary hosts of henipaviruses are fruit bats (family Pteropodidae) in Africa, Asia, and Oceania (*5*). Although Nipah virus causes no apparent disease in bats (*6*,*7*), the case-fatality rate in humans can be 40%–70% (*2*,*8*,*9*). In addition, Nipah virus has characteristics that enable repeated human outbreaks. Its bat hosts are widespread in South Asia and Southeast Asia, regions with dense human and livestock populations (*10*), which could lead to virus spillover and spread (*11*). Nipah virus can transmit directly from bats when humans consume date palm sap that is contaminated with bat saliva, urine, or feces or can transmit indirectly through spillover to domesticated animals (*12*–*14*). 

Since 2001, Bangladesh has experienced multiple Nipah virus outbreaks with confirmed person-to-person transmission, albeit below the threshold necessary for sustained epidemics (*8*); however, the virus transmitted rapidly among pig populations in Malaysia, producing infection rates of 100% on some farms, and spread between farms through shipments of infected animals (*15*,*16*). No commercially available vaccines or therapeutics for Nipah virus exist to prevent or mitigate disease in case of an epidemic, although these interventions are areas of active research (*17*,*18*). Finally, RNA viruses such as Nipah have high mutation rates, which are a predictor of zoonotic potential (*19*). Although documented genetic diversity within Nipah viruses is limited (*20*–*24*), high mutation rates could potentially produce variants with sufficient transmissibility in humans to cause a sustained epidemic (*25*,*26*). Given the wide geographic range and unsampled diversity of Nipah viruses, variants that are more transmissible among humans might exist and circulate in bats, and each spillover event could be an opportunity for such variants to emerge (*27*).

Genetic and phenotypic diversity among Nipah viruses exists, but the human health implications are unclear. Nipah virus genotypes from Bangladesh and India are genetically distinct from genotypes from Malaysia (*22*–*24*). Although Malaysia genotypes are less diverse than those from Bangladesh and India (*24*), genotypes from Malaysia derive solely from pigs, humans, and bats during the 1998–1999 outbreak, whereas genotypes from Bangladesh and India derive from multiple human outbreaks and surveys of bats since 2004. Another difference is that person-to-person transmission of Nipah virus has rarely been observed in Malaysia (*28*–*30*) but accounted for one third of reported cases in Bangladesh (*8*) and >75% of cases in India (*1*,*9*,*31*). However, person-to-person transmission in Malaysia was not investigated beyond healthcare workers, and <10% of persons with Nipah virus transmit it to another person, usually a family caregiver (*8*,*28*). Some of this variation in transmission mode and severity could reflect differences in exposure, sampling, infrastructure, and culture between countries, but differences between viral strains might explain additional variation. Case-patients in Malaysia were less likely to experience cough, difficulty breathing, or abnormal chest radiography than case-patients in Bangladesh (*29*,*32*,*33*). These differences in transmissibility and pathogenicity between Nipah virus strains from Malaysia and Bangladesh have been observed in some animal experiments, although with conflicting results (*34*–*36*). The reviewed evidence suggests that genetic variation in Nipah virus might produce differences in pathogenicity or transmissibility, so more transmissible strains of Nipah virus could be circulating undetected in bat populations.

Knowledge of Nipah virus diversity is limited to the few virus sequences obtained to date. Available sequences from GenBank and recent studies (*20*,*24*) include only 76 Nipah virus genomes, 51 of which derive from human patients, and 153 nucleocapsid protein genes, 37 of which derive from humans. Previous studies have not been optimized to characterize Nipah virus genotypes circulating in bats. 

The Indian flying fox (*Pteropus medius*) is the major reservoir of Nipah virus in Bangladesh and India (*37*,*38*). Longitudinal surveys indicate that exposure to Nipah virus is high (≈40%) in some *P. medius* populations in Bangladesh on the basis of serologic tests, but the prevalence of detectable Nipah virus RNA is low (<5%) at any given time (*37*). In addition, viral loads in collected bat samples are often low (*24*), limiting the success of virus sequencing or isolation necessary for describing viral diversity. Sampling methods that increase the success of detecting Nipah virus in bats and increase yield so that sequencing is possible would be useful for monitoring genetic changes in this virus. In this study, we focused Nipah virus detection to *P. medius* bat roosts near human cases identified in Bangladesh during outbreak investigations during 2012–2019. We aimed to identify whether bat roosts were actively shedding Nipah virus RNA in urine and how long shedding continued after initial detection. In addition, we sought to identify characteristics of bat roosts potentially associated with higher likelihood of testing positive.

## Materials and Methods

### Nipah Virus Case Investigations

Human case-patients with suspected Nipah virus infection with a history of consuming date palm sap were identified at 3 surveillance hospitals in the Faridpur, Rajshahi, and Rangpur Districts of Bangladesh (*39*). Additional suspected cases in other regions were identified from media reports (*40*). A total of 47 primary cases of Nipah virus representing spillover from bats were identified in 2012–2018; we investigated 17 in this study. Four additional spillover cases were investigated in 2019, but the total number of spillover cases from that year is unclear because of a lack of reporting. Case exposure to Nipah virus was evaluated with ELISA or PCR (*41*). Investigation teams visited the suspected case villages to gather evidence of case clusters and identify the exposure route (*42*). In some cases, teams were deployed before human cases were confirmed by ELISA or PCR.

Teams searched for *P. medius* bat roosts within a 20 km radius of the human case-patient’s residence by asking community members about known roost sites and by scouting. Some identified roosts were located on burial grounds or over water and could not be sampled (Appendix 1 Table 1). During 12:00–4:00 AM, teams placed 4–20 polyethylene tarps under each roost, depending on the available area and size of the roost, to collect urine. Tarps were concentrated under branches with denser aggregations of bats. Tarps were ≈6 feet × 4 feet in size before 2019 and 3 feet × 2 feet in 2019; we made this change so that fewer bats contributed to urine pools to improve estimates of prevalence (*43*). During 5:00–6:00 AM, teams returned to the roosts and collected bat urine from the tarps with a sterile syringe. Urine collected from tarps was either pooled by individual tarp or mixed together from multiple tarps and then divided into aliquots. We found no significant difference in Nipah detection between the 2 strategies (Appendix 1). We tested aliquots for Nipah virus RNA at icddr,b (Dhaka, Bangladesh) or National Institutes of Health (Hamilton, MT, USA) laboratories by using quantitative real-time reverse transcription PCR (qRT-PCR) targeting the nucleoprotein gene (*44*). Roosts with Nipah virus RNA detected in any aliquots at the first sampling event were revisited (3–16 days between sampling events) until all aliquots from a roost tested negative. Attempts to culture Nipah virus from positive samples at National Institutes of Health yielded no virus isolates; viral culture was not attempted at icddr,b because of the absence of BioSafety Level 4 facilities.

### Statistical Analysis

For each laboratory-confirmed spillover case of Nipah virus in a human, we recorded the symptom onset date and the coordinates of the case-patient’s residence. Teams identified the probable date of patient exposure to Nipah virus by the date of palm sap consumption for some cases; otherwise, the exposure date was assumed to be 7 days before symptom onset on the basis of the mean incubation period of Nipah virus for primary cases linked to spillover (*45*).

We used logistic regression to assess features of the roost sites associated with a roost testing positive for Nipah virus at the first sampling visit. Covariates in the model included the number of days between the first case-patient exposure to date palm sap and roost sampling, the number of bats in the roost, the distance between the case-patient’s home and the roost site, and the number of human spillover cases associated with each nearby roost. We then performed model selection to choose important features using Akaike corrected information criterion (*46*).

For all roost sites that tested positive for Nipah virus at first sampling, we recorded the number of tested urine aliquots that were positive for Nipah virus at each visit. Because cycle threshold (Ct) values from qRT-PCR were not reported for all tests, we used the proportion of positive aliquots as a proxy for the intensity of virus shedding in bats, assuming that roosts with higher virus concentrations in urine would produce more positive aliquots. We then analyzed changes in the proportion of positive aliquots across roosts along 2 time axes. We aligned dates to the number of days since the presumed exposure date of the first human spillover Nipah case associated with each roost site. We then aligned roost-sampling dates to the number of days since the start of the calendar year for comparison. We fit binomial linear models to estimate the probability of detecting a Nipah virus–positive aliquot at each roost along each time axis.

To evaluate the utility of sampling bat roosts near human Nipah virus cases as a surveillance approach, we compared the rate of successful Nipah virus detections from this study to data reported by Epstein et al. (*37*). Samples from that study were collected quarterly from a *P. medius* bat roost in Faridpur District during 2007–2012 as part of a longitudinal study; from visits to different roosts throughout Bangladesh during 2006–2011 as part of a cross-sectional spatial analysis; or as part of Nipah virus outbreak investigations in 2009, 2010, and 2012. Urine samples were either collected from individual bats or from underneath roosts. For these comparisons, we considered each roost visit as a discrete sampling event, including repeat visits to the same roost. Ignoring the initial visits to 7 roosts near 5 suspected human cases that were Nipah virus–negative, the 23 roosts in our study were sampled across 47 visits. We made comparisons between studies for the number of sampling visits with positive Nipah detections and the number of positive urine samples (individual or pooled aliquots from roosts) across all sampling visits or during the first visit to each roost. We evaluated comparisons by using a χ^2^ test of proportions or Fisher exact test. We considered statistical tests significant if p values were <0.05.

### Ethics

All study participants or proxies provided informed consent before participation and personally identifiable information from patients was delinked from the data before use. Written permission was obtained from the Bangladesh Forest Department for sampling the bats, and team members obtained permission from landowners before sampling roosts. Protocols for case investigations and roost sampling were reviewed and approved by the Institutional Review Board at icddr,b.

## Results

**Figure 1:**
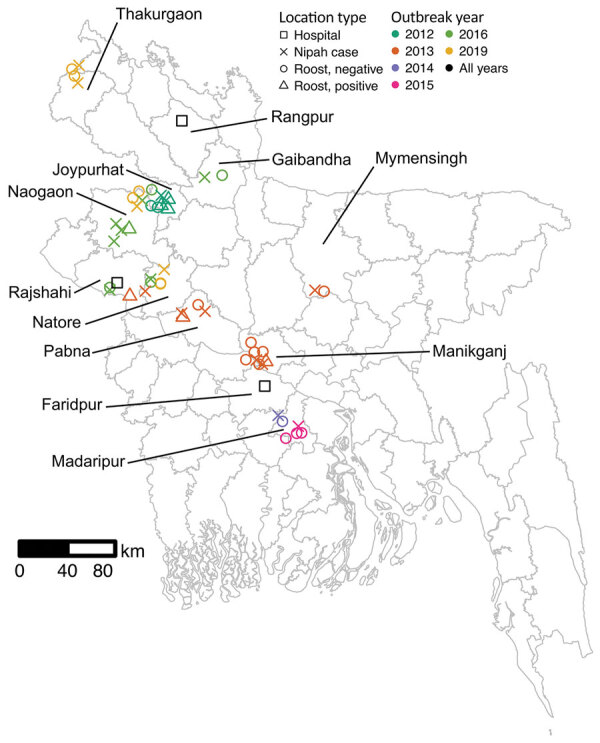
Locations of human Nipah cases (n = 21) and *Pteropus medius* bat roosts (n = 30) investigated in Bangladesh, 2012–2019. Roosts with urine aliquots that tested positive for Nipah virus RNA at the first sampling visit are indicated with triangles. Points have been jittered a small amount to increase visibility. Districts with human Nipah virus cases, identified bat roosts, or Nipah surveillance hospitals are labeled.

Teams investigated roosts near homes of 21 suspected human cases of Nipah virus infection during 2012–2019 (Appendix 1 Table 1). The cases were clustered in the central and northwest districts of Bangladesh, close to the 3 surveillance hospitals (Figure 1). Symptom onset for patients occurred in winter (December–February), with the exception of 1 case-patient in Manikganj District whose symptoms began in March 2013. No roost investigations were performed in 2017 and 2018 because of funding constraints.

For each case-patient, we identified 1–3 *P. medius* bat roosts within 0–17.9 km of the patient’s home (Appendix 2 Table 1). An additional 5 identified roosts were not sampled because they were located on burial grounds or over water (Appendix 1 Table 1). We sampled a total of 30 roosts. The first sampling visits occurred 17–62 days after the case-patients’ exposure to date palm sap, either reported from the case investigation or back-calculated as 7 days before the onset of symptoms (Appendix 2 Table 1). Five of the suspected patients tested negative for Nipah virus by ELISA or PCR, and the 7 roosts identified near the patients’ homes yielded no Nipah virus RNA. Because our interest was in whether sampling near human Nipah virus cases would help to identify roosts with active Nipah virus shedding, we excluded suspected but Nipah virus–negative case-patients and associated bat roosts from statistical analyses. Sensitivity analyses that included these samples produced statistically similar results. Testing by qRT-PCR of pooled urine aliquots detected 7/23 (30%) roosts as positive for Nipah virus RNA in >1 aliquots at the first sampling visit.

**Figure 2:**
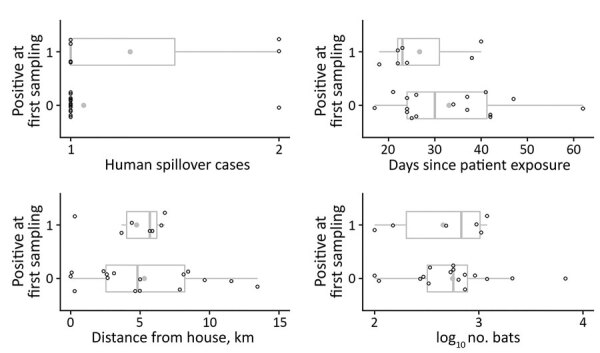
Descriptive variables for 23 *Pteropus medius* bat roosts sampled near confirmed human Nipah virus cases, Bangladesh, 2012–2019. Open circles show the values associated with the first human case associated with each roost; gray circles indicate means for each variable and positivity status (0 or 1). Vertical lines within boxes indicate medians; box left and right edges indicate the 25th and 75th percentiles; error bars indicate +1.5 times the interquartile range.

We performed Logistic regression on the presence of Nipah virus RNA in roost urine at the first sampling event on 22 distinct roosts using 4 explanatory variables; 1 roost was omitted because of missing data on the number of bats. Roosts with positive urine aliquots tended to have more associated human Nipah spillover cases, were sampled sooner after patient exposure, were more distant from patients’ homes, and had a smaller number of bats, but none of these variables were significantly associated with roost positivity in univariate or multiple regression analyses (Figure 2; Appendix 1 Table 2), and Akaike corrected information criterion identified the intercept-only model as the best model (Appendix 1 Table 3).

**Figure 3:**
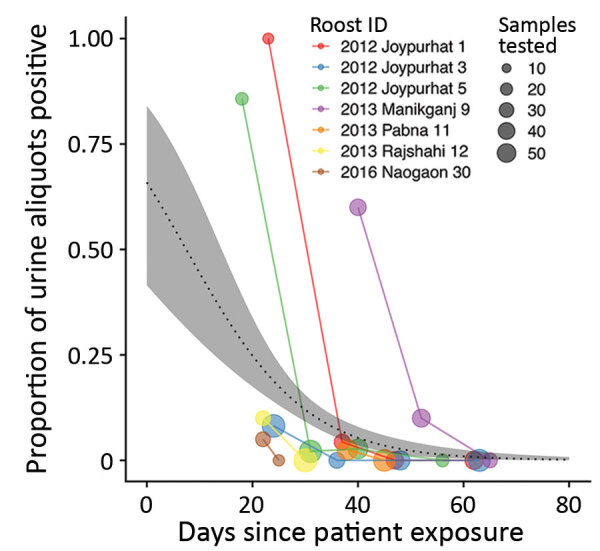
Results of screening of *Pteropus medius* bat roost urine aliquots for Nipah virus RNA, Bangladesh, 2012–2019. For each roost, the proportion of urine aliquots out of the total tested (indicated by the size of the circles) is aligned along a time axis of days since the first associated case-patient was exposed to Nipah virus in date palm sap. Time since patient exposure was either reported during the investigation or back-calculated as 7 days before reported symptom onset.

For the 7 roosts where Nipah virus RNA was detected >1 time, data were compiled on the number of urine aliquots that tested positive at each repeated sampling visit. Of these 7 roosts, 4 were positive at the first visit only and were revisited only once. The other 3 roosts remained positive at 1–2 additional sampling visits, although the proportion of aliquots that tested positive declined rapidly with the time since exposure of the first associated human case (Figure 3). For the 2 roosts with reported Ct values from qRT-PCR, the proportion of positive aliquots decreased over the repeated sampling visits while Ct values increased, indicating a decline in viral load (Appendix 1 Table 4).

Fitting a binomial model to the PCR data predicted that the probability of detecting at least 1 urine aliquot from under-roost sampling as positive for Nipah virus RNA at the time the associated case-patient was presumably exposed (day 0) was 0.66 (95% CI 0.42–0.84) (Figure 3). This probability declined to 0.02 (95% CI 0.01–0.04) by day 52, when the last positive roost aliquots were detected, and to 0.01 (95% CI 0–0.02) by day 65, when the last roost was sampled. We also fit a binomial model by using the days elapsed since the start of the calendar year (Appendix 1 Figure), but alignment of the virus detections among the roosts was less clustered on that time axis than the days-since-patient-exposure time axis, and the binomial model did not show a significant trend in detection over time.

Roost urine samples from our study and individual urine samples from longitudinally sampled roosts in Epstein et al. (*37*) produced similar proportions of positive sampling visits (comparison A in Table); the detection rate was also similar if only the first visit to each roost in our study was considered (7/23, 30%). In contrast, the proportion of positive aliquots from all sampling visits was significantly higher in our investigations than in the individual urine samples from longitudinal roosts in Epstein et al. (*37*) (comparison B in Table). The detection rate from our study for positive urine aliquots at the first sampling visit was also higher than the detection rate for individual urine samples collected from 8 roosts from a cross-sectional study by Epstein et al. (*37*) (comparison C in Table). The detection rate for positive urine aliquots from our study was substantially higher than the detection rate from similarly pooled urine aliquots from underneath longitudinal and cross-sectional roosts in Epstein et al. (*37*) (comparison D in Table). Last, outbreak investigations of roosts performed by Epstein et al. (*37*) produced a higher detection rate than our own roost investigations (comparison E in Table), although only 4 roosts were visited by Epstein et al. (*37*), and the same roosts were not repeatedly visited as we did in our study.

**Table:** Nipah virus detection success from study of bat roosts after spillover events, Bangladesh, 2012–2019, compared with results from previous study*

Test ID	Data from this study			Data from Epstein et al. (*37*)		Statistical test results
	Description	No. positive/no tested (%)		Description	No. positive/no tested (%)	
A	Positive sampling visits based on pooled roost urine aliquots where >1 urine aliquot tested positive†	11/47 (23%)		Positive sampling visits based on individual urine samples from longitudinal roosts where >1 individual urine sample tested positive	5/18 (28%)	OR = 0.84,‡ p = 0.76
B	Positive roost urine aliquots from sampled roosts across 47 sampling visits†	51/1,042 (4.9%)		Positive individual urine samples from longitudinal roosts across 18 sampling visits	8/1,671 (0.48%)	χ^2^ = 56.8, p<0.001
C	Positive roost urine aliquots from the first visit to 23 sampled roosts†	45/525 (8.6%)		Positive individual urine samples from 8 roosts from a cross-sectional spatial study across districts of Bangladesh	0/555 (0%)	χ^2^ = 47.5, p<0.001
D	Positive roost urine aliquots from sampled roosts across 47 sampling visits†	51/1042 (4.9%)		Positive roost urine aliquots from longitudinal roosts and cross-sectional roosts, excluding samples from outbreak investigations	2/725 (0.28%)	χ^2^ = 29.8, p<0.001
E	Positive roost urine aliquots from sampled roosts across 47 sampling visits†	51/1042 (4.9%)		Positive roost urine aliquots from outbreak investigations, n = 4	19/104 (18.3%)	χ^2^ = 27.2, p<0.001

*ID, identification; OR, odds ratio. †Excludes the 7 roosts associated with 5 human cases that initially tested negative for Nipah virus. Statistical tests that included these samples produced similar results. ‡By Fisher exact test.

## Discussion

Nipah virus spillover from bats occurs sporadically in Bangladesh, so surveillance that optimizes viral detection in bats is a challenge. In contrast with cross-sectional or longitudinal bat roost surveillance used previously (*37*), the roost sampling in this study was triggered by Nipah virus outbreaks in nearby villages. Our approach identified roosts with active Nipah virus shedding at an equivalent rate to background surveillance (*37*) but had a higher detection rate in roost urine on a per sample basis. These results indicate that investigating roosts near spillover cases is more efficient than cross-sectional or longitudinal surveillance for obtaining samples with detectable viral RNA (Table). Repeated visits to positive roosts also demonstrated that viral RNA was detectable for weeks after the purported exposure date of human cases, although the proportion of positive urine aliquots declined sharply with time. Detections by PCR do not always produce sequences or genomes, so surveillance approaches that increase the number or quality of detections (e.g., higher viral loads) could maximize opportunities to collect samples with sufficient viral RNA for sequencing. These data suggest that rapid investigations to sample urine from bat roosts could increase the probability of detecting and sequencing Nipah virus. Used in combination with longitudinal sampling of roosts and surveillance of human or domesticated animal cases, this method could enhance our understanding of Nipah virus dynamics and genetic diversity in bats.

This study also provides critical information about the timing of Nipah virus shedding in bats in Bangladesh. Longitudinal surveys have shown that Nipah virus shedding from bats is sporadic throughout the year (*37*), so the peaks in viral detection in roost urine from our study likely coincided with shedding events. However, because these shedding events occurred during winter (when date palm sap is harvested for human consumption), bat visits to date palm trees might be more likely to contaminate sap with virus and lead to human infections (*47*). This factor suggests that the intensity of shedding events in bats occurring in winter could help to explain some of the spatiotemporal variation in the number of human spillovers that occur in Bangladesh annually (*42*), although more data on the frequency and timing of shedding events and human sap consumption will be needed to fully understand the dynamics of Nipah virus spillover.

Our findings come with several caveats because of limitations in our sample size and study design. Our analysis of factors associated with a roost testing positive at first sampling was unable to pinpoint significant relationships, likely because of low statistical power. We also did not systematically attempt virus isolation or sequencing in all positive samples, so we cannot estimate the probability of successful isolation or sequencing. However, Nipah virus isolates and sequences have been obtained from some of the roost urine samples included in this study. One of the positive roosts in Joypurhat from 2012 produced 9 nucleocapsid sequences (GenBank accession nos. MT890702–10) (*24*), and the positive roost in Manikganj from 2013 produced 10 virus isolates with full-genome sequences (GenBank accession nos. MK575060–9) (*21*). In fact, of the 39 Nipah virus sequences from bats in Bangladesh, 28 (72%) came from under-roost urine samples and 24 (86%) came from roost investigations near human cases (Appendix 2 Table 2). These patterns suggest that roost urine, especially from roosts near human spillover cases, might contain sufficient Nipah virus for sequencing or culture. Furthermore, in several human case-patients in Joypurhat in 2012 who drank date palm sap, we identified Nipah virus sequences that were genetically similar (>99.6% sequence identity) to sequences from the Joypurhat bat roost (roost 1 in Figure 3), providing additional evidence that connects virus shedding in local bat populations with human cases (Appendix 1). Future investigations could track how viral load in roost urine varies during viral shedding events, which could improve sequencing and isolation success and shed light on the ecologic conditions that lead to Nipah shedding from bats (*48*).

Our case investigations were also limited to the catchment area of 3 surveillance hospitals and the winter seasonality of Nipah virus spillover surveillance. This design systematically misses virus shedding events at bat roosts outside the surveillance area or during seasons when humans are not drinking fresh date palm sap (*13*). The logistical constraints of our surveillance approach cannot capture all Nipah virus genotypes circulating in *P. medius* across Bangladesh, but increasing the number of detections is still crucial, especially given the few Nipah virus isolates currently available (n = 11). Reactive roost investigations could be complemented with additional roost surveys outside of surveillance areas to learn more about Nipah virus transmission and genetic diversity in bat populations across Bangladesh.

This study provides proof of concept that reactive investigations of bat roosts near human Nipah virus cases can complement ongoing surveillance efforts and could increase the likelihood of viral detection and sequencing. Improvements in virus detection would aid in characterizing the genetic diversity of Nipah viruses circulating in bats and identify novel genotypes that might pose pandemic threats. Furthermore, these data provide evidence that viral shedding can continue for weeks after an initial spillover event, posing a hazard for additional contamination. Precise knowledge of when bats are shedding Nipah virus could be used to deploy public health campaigns more efficiently, such as by using barriers to prevent bat access to date palm sap (*49*).

Appendix 1 Additional information about Nipah virus detection at bat roosts after spillover events, Bangladesh, 2012–2019

Appendix 2 Additional data used in study of Nipah virus detection at bat roosts after spillover events, Bangladesh, 2012–2019
