# Supplementary material for: Nipah Virus Detection at Bat Roosts after Spillover Events, Bangladesh, 2012–2019
Source: Emerg Infect Dis. 2022 Jul;28(7):1384–92. doi: 10.3201/eid2807.212614 (PMC9239894; doi:10.3201/eid2807.212614)
Supplement: Appendix 1 — Additional information about Nipah virus detection at bat roosts after spillover events, Bangladesh, 2012–2019 [file 21-2614-Techapp-s1.pdf]

Article DOI: https://doi.org/10.3201/eid2807.212614

Nipah Virus Detection at Bat Roosts after Spillover Events, Bangladesh, 2012–2019

Appendix 1

Supplemental Materials and Methods

Pooling of Roost Urine

Collected urine from underneath roosts was aggregated in 50 mL Falcon tubes from tarps either individually (i.e., one tube per tarp) or mixed together from all tarps and then divided into aliquots for testing: 0.25–0.3 ml of urine in 0.75–1.0 ml of viral transport medium (VTM) or lysis buffer before 2014, or equal volumes (0.1–0.3 ml) of urine and media after 2014. The two tarp pooling strategies were used at different sampling events throughout the period of the study. According to our records, 22 sampling events pooled urine from all tarps together and 24 events pooled urine individually by tarp (the pooling strategy for one event could not be determined from field records). For three sampling events where urine was pooled individually by tarp, multiple aliquots from different tarps were positive for Nipah RNA by PCR. Additionally, for the sampling events where urine was pooled from all tarps, the proportion of PCR positive aliquots ranged from 0%–100%. We did not observe a clear pattern that indicated that the large pooled volume of urine in these sampling events strictly prevented viral detection due to dilution of viral RNA (i.e., all aliquots were negative or only a few were positive) or that aggregating urine across pools led to saturation (i.e., all aliquots were positive). We could not detect a significant difference between the two pooling strategies in terms of the proportion of positive sampling events: 7/22 (32%) for events with all tarps mixed together versus 4/24 (17%) for events with separate pools per tarp (Fisher's test odds ratio = 1.89, p = 0.5). We also did not detect a significant difference between the strategies in terms of the proportion of positive aliquots across all sampling events: 30/527 (5.7%) for events with all tarps mixed together versus 21/499 (4.2%) for events with separate pools per tarp ($\chi^2 = 0.9$, $p = 0.34$). Therefore, we concluded that these differences in pooling strategy did not appear to interfere with our ability to detect Nipah RNA in roost urine and were suitable to be analyzed together.

Epidemiologic Links between Nipah Virus Sequences in Humans and Bats

The genetic similarity between Nipah viruses found in humans and those detected circulating in bat roosts can provide additional data supporting a connection between a shedding event in bats to human spillovers. Although this was not a primary focus of this study, connections from the available data are provided below. Because not all human cases are confirmed by PCR, cases with PCR positives do not always produce a Nipah virus sequence or genome (due to low viral loads), and only a subset of all cases were included in the investigations in this study, there were only four human cases included in this study that have available nucleocapsid sequences or genomes (*1,2*) (Appendix 2 Table 2). Likewise, only two of the bat roosts investigated in this study produced nucleocapsid sequences: roost #1 in Joypurhat in 2012 and roost #9 in Manikganj in 2013. These were two of the roosts with the highest number of urine aliquots positive at the first sampling event (Figure 3 in the main article, https://wwwnc.cdc.gov/EID/article/28/7/21-2614-F3.htm). None of the other roosts investigated during the study produced any sequences, presumably because of low viral loads, as indicated by the few aliquots positive for these roosts (*1,3*) (Appendix 2 Table 2).

The only roost where sequence data was obtained from both human cases and the bat roost was from roost #1 in Joypurhat in 2012; no Nipah sequences were available for the human cases from Manikganj in 2013. As indicated by Rahman et al. (*1*) in their analysis of human and bat nucleocapsid sequences, two human cases from Joypurhat (RS90412 and RS90612, GenBank accession numbers MT890728 and MT890729) that drank date palm sap together had sequences that closely clustered with sequences from the Joypurhat bat roost #1 (samples J101–J107, nucleocapsid GenBank accession numbers MT890702–MT890708); case RS90412 is case JP00112 in our dataset. Using the NCBI Basic Local Alignment Search Tool (BLAST) (*4*), both of these sequences share >99.6% nucleocapsid sequence identity with sequences J101–J107 from the Joypurhat bat roost. A full genome from an additional human case from the 2012 Joypurhat cluster was sequenced by Whitmer et al. (*2*), case ID BG00112 in our dataset, GenBank accession number MK673579. A BLAST search indicates that the nucleocapsid sequence from this genome shares >99.7% sequence identity with the J101–J107 nucleocapsid sequences from the Joypurhat #1 roost. This suggests that the human cases in Joypurhat were infected with Nipah virus quasispecies that were highly similar to those detected in the nearby bat roost. The human cases reportedly drank date palm sap (*1*), and it is known from other studies that *Pteropus* bats

frequently drink date palm sap during winter months (*5*,*6*), so date palm sap was the most likely vector that connected the virus shedding event occurring in the Joypurhat bat population to the spillover cases. The Nipah sequences from human cases and the bat roost in Joypurhat are highlighted in red in Appendix 2 Table 2.

References

1. Rahman MZ, Islam MM, Hossain ME, Rahman MM, Islam A, Siddika A, et al. Genetic diversity of Nipah virus in Bangladesh. Int J Infect Dis. 2021;102:144–51. PubMed https://doi.org/10.1016/j.ijid.2020.10.041

2. Whitmer SLM, Lo MK, Sazzad HMS, Zufan S, Gurley ES, Sultana S, et al. Inference of Nipah virus evolution, 1999–2015. Virus Evol. 2021;7:veaa062. **PMID 34422315**

3. Anderson DE, Islam A, Crameri G, Todd S, Islam A, Khan SU, et al. Isolation and full-genome characterization of Nipah viruses from bats, Bangladesh. Emerg Infect Dis. 2019;25:166–70. PubMed https://doi.org/10.3201/eid2501.180267

4. Johnson M, Zaretskaya I, Raytselis Y, Merezhuk Y, McGinnis S, Madden TL. NCBI BLAST: a better web interface. Nucleic Acids Res. 2008;36(Web Server):W5-9. PubMed https://doi.org/10.1093/nar/gkn201

5. Khan MSU, Hossain J, Gurley ES, Nahar N, Sultana R, Luby SP. Use of infrared camera to understand bats' access to date palm sap: implications for preventing Nipah virus transmission. EcoHealth. 2010;7:517–25. PubMed https://doi.org/10.1007/s10393-010-0366-2

6. Islam A, McKee C, Ghosh PK, Abedin J, Epstein JH, Daszak P, et al. Seasonality of date palm sap feeding behavior by bats in Bangladesh. EcoHealth. 2021;18:359–71. PubMed https://doi.org/10.1007/s10393-021-01561-9

Appendix 1 Table 1. *Pteropus medius* roosts identified and sampled near suspected human index cases of Nipah virus infection in Bangladesh, 2012–2019*

Year	Division	District	Suspected Nipah virus cases	Total identified roosts	Sampled roosts	Unsampled roosts
2012	Rajshahi	Joypurhat	2	6	5	1
2013	Mymensingh	Mymensingh	1	1	1	0
	Dhaka	Manikganj	1	3	3	0
	Rajshahi	Pabna	2	2	2	0
		Rajshahi	1	1	1	0
2014	Dhaka	Madaripur	1	1	1	0
		Manikganj	1	3	3	0
2015	Dhaka	Madaripur	1	3	3	0
2016	Rajshahi	Joypurhat	1	1	1	0
		Naogaon	3	1	1	0
		Natore	1	1	1	0
		Rajshahi	1	1	1	0
	Rangpur	Gaibandha	1	1	1	0
2019	Rajshahi	Naogaon	1	2	2	0
		Natore	1	5	2	3
		Rajshahi	1	2	1	1
	Rangpur	Thakurgaon	1	1	1	0

*Roosts that were identified but not sampled were not accessible because they were located on burial grounds or over water.

Appendix 1 Table 2. Multivariate logistic regression model coefficients for the presence of Nipah virus RNA at *Pteropus medius* roosts (n = 22) near human cases

Variable	Description	Coefficient (odds)	z-value	p-value
(Intercept)		0.23	−0.53	0.6
Bats	Number of bats in roost	1	−1.2	0.23
Days	Days elapsed between the first case exposure and roost sampling	0.91	−1.3	0.2
Distance	Distance (km) between the case house and the roost	0.87	−0.74	0.46
Cases	Number of human spillover cases associated with each sampled roost	109	1.8	0.075

Appendix 1 Table 3. Model selection for variables associated with the presence of Nipah virus RNA at *Pteropus medius* roosts (n = 22) near human cases*

Model	df	AICc	ΔAICc
(Intercept)	1	27.98	0
Cases	2	28.01	0.03
Bats + cases	3	28.33	0.35
Cases + days	3	28.78	0.8
Days	2	29.02	1.04
Bats + cases + days	4	29.5	1.51
Bats	2	30.03	2.05
Distance	2	30.4	2.42
Cases + distance	3	30.67	2.69
Bats + cases + distance	4	31.17	3.19
Bats + days	3	31.26	3.28
Cases + days + distance	4	31.5	3.57
Days + distance	3	31.71	3.73
Bats + cases + days + distance	5	32.3	4.32
Bats + distance	3	32.73	4.75
Bats + days + distance	4	34.16	6.18

*AICc, Akaike corrected information criterion; ΔAICc, difference in AICc value relative to the top model (lowest AiCc); df, degrees of freedom.

Appendix 1 Table 4. Changes in the proportion of urine aliquots testing positive over repeated visits and associated Ct values from qRT-PCR

Roost	Sampling date	Urine aliquots positive	Average Ct value
2013 Manikganj 9	11 April 2013	15/25 (60%)	30.5
	23 April 2013	3/30 (10%)	36.8
	6 May 2013	0/20 (0%)	ND
2013 Rajashahi 12	1 February 2013	2/20 (10%)	38.6
	9 February 2013	0/50 (0%)	ND

*Ct, cycle threshold; qRT-PCR, quantitative real-time reverse transcription PCR.

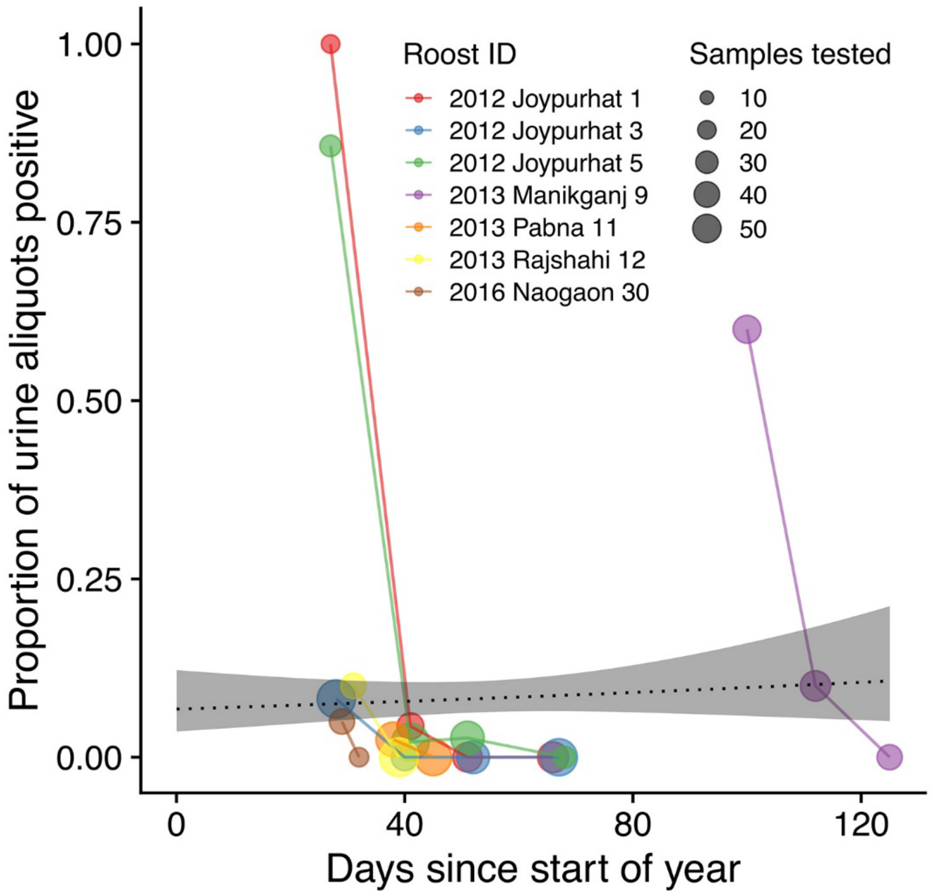

Appendix 1 Figure. Results of screening *Pteropus medius* roost urine aliquots for Nipah virus RNA. For each roost, the proportion of urine aliquots out of the total tested (shown by the size of points) is aligned along a time axis of the days since the start of the calendar year for each roost investigation.
